# Supplementary figures and images for: Exploring turn demands of an English Premier League team across league and knockout competitions over a full season
Source: PLoS One. 2025 Apr 23;20(4):e0321499. doi: 10.1371/journal.pone.0321499 (PMC12017906; doi:10.1371/journal.pone.0321499)

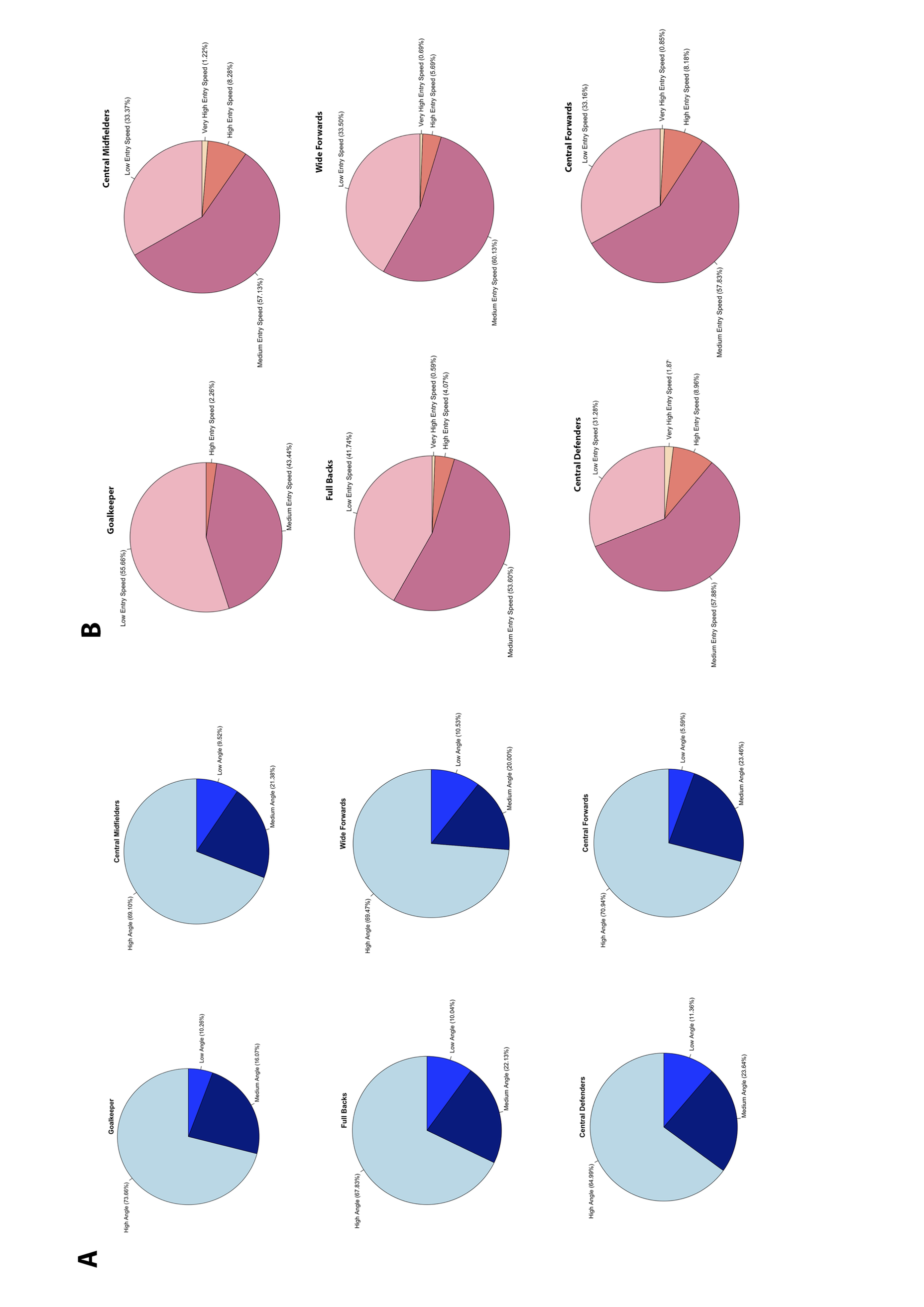

Supplement: S1 Figure — The proportion of turns for each position can be seen as follows: Goal Keepers; Full Backs; Central Defenders; Central Midfielders; Winger Forwards; Central Forward. (TIF) [file pone.0321499.s001.tif]

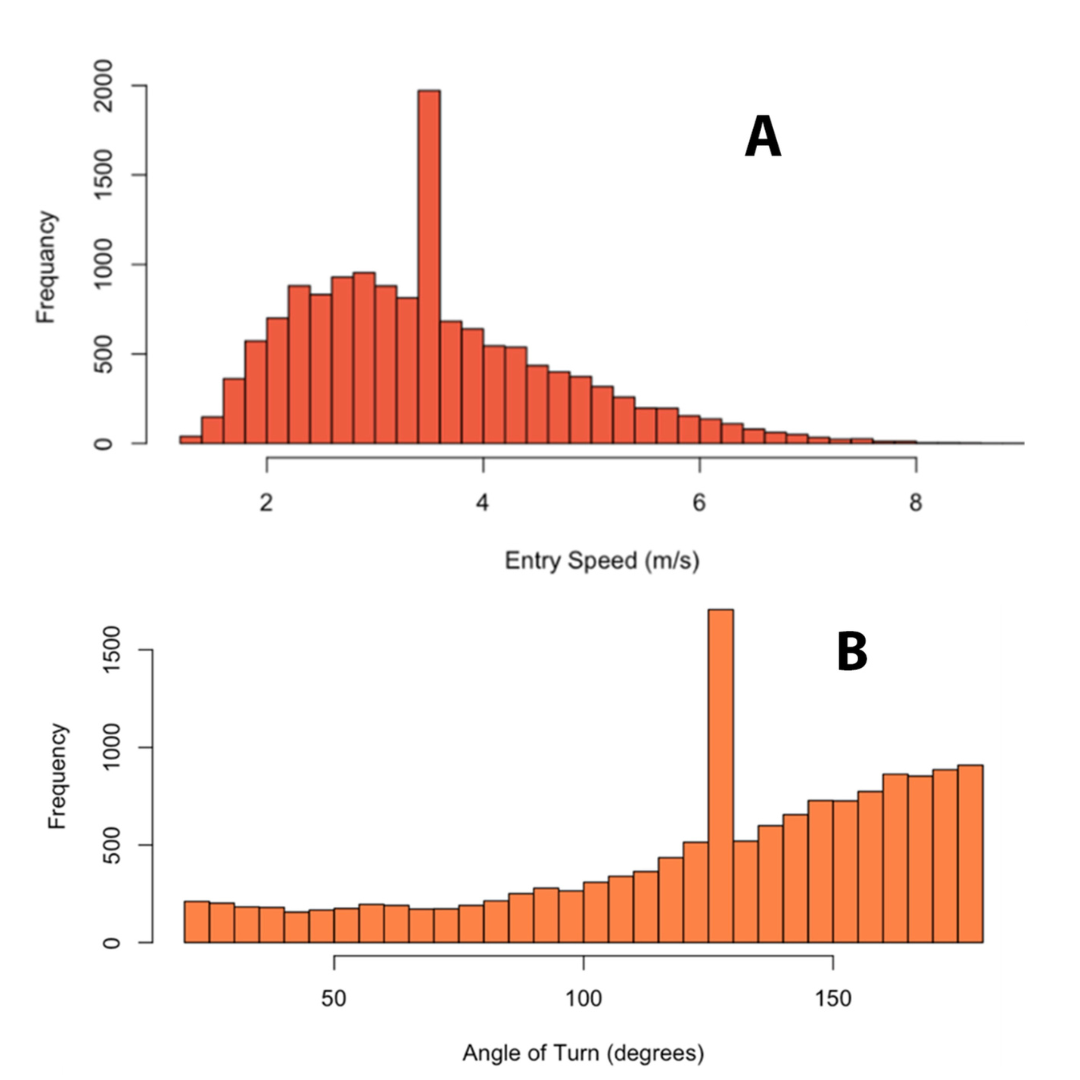

Supplement: S2 Figure — (TIF) [file pone.0321499.s002.tif]
