# Supplementary material for: Exploring turn demands of an English Premier League team across league and knockout competitions over a full season
Source: PLoS One. 2025 Apr 23;20(4):e0321499. doi: 10.1371/journal.pone.0321499 (PMC12017906; doi:10.1371/journal.pone.0321499)
Supplement: S3 Table — Inter-quartile range (IQR). 95% Confidence Intervals. GK: Goalkeeper. FB: Full-back. CD: Central Defender. CM: Central Midfielder. WF: Winger Midfielder. CF: Central Forward. (DOCX) [file pone.0321499.s003.docx]

| **Position** | **Low Angle (Mean ± SD, IQR)(95% CI)** | **Medium Angle (Mean ± SD, IQR) (95% CI)** | **High Angle (Mean ± SD, IQR) (95% CI)** | **Total Turns (Mean ± SD, Min–Max) (95% CI)** |
| --- | --- | --- | --- | --- |
| **GK** | 1.44 ± 0.73 (1.00) (0.29 to 2.59) | 1.57 ± 1.16 (1.00) (−1.31 to 4.45) | 3.59 ± 2.47 (2.00)(−2.56 to 9.74) | 4.77 ± 3.32 (1–15) (−3.50 to 12.04) |
| **FB** | 2.88 ± 1.69 (2.00) (1.12 to 4.64) | 6.05 ± 3.50 (4.75) (1.72 to 10.38) | 18.10 ± 7.65 (10.00) (8.60 to 27.60) | 26.36 ± 10.32 (2–51) (13.57 to 39.15) |
| **CD** | 3.03 ± 1.76 (2.00) (1.01 to 5.05) | 5.47 ± 2.50 (3.00) (1.49 to 9.45) | 15.03 ± 8.31 (6.00) (1.80 to 28.26) | 22.82 ± 9.41 (1–54) (7.83 to 37.81) |
| **CM** | 3.38 ± 1.96 (2.00) (1.98 to 4.78) | 7.38 ± 3.48 (5.00) (4.47 to 10.29) | 23.86 ± 15.72 (11.00) (10.71 to 37.01) | 34.52 ± 17.93 (3–102) (19.52 to 49.52) |
| **WF** | 2.48 ± 1.56 (2.00) (0.81 to 4.15) | 4.40 ± 2.36 (3.00) (1.93 to 6.87) | 14.38 ± 8.98 (8.75) (4.94 to 23.82) | 20.70 ± 11.18 (2–68) (6.87 to 34.53) |
| **CF** | 1.71 ± 0.95 (1.50) (0.13 to 3.29) | 7.00 ± 1.15 (1.50) (4.14 to 9.86) | 20.57 ± 3.51 (5.50) (11.84 to 29.30) | 29.83 ± 4.49 (25–37) (20.77 to 38.89) |
| **Overall** | 2.92 ± 1.79 | 5.82 ± 3.35 | 16.84 ± 12.14 | 24.50 |
