# Supplementary material for: Exploring turn demands of an English Premier League team across league and knockout competitions over a full season
Source: PLoS One. 2025 Apr 23;20(4):e0321499. doi: 10.1371/journal.pone.0321499 (PMC12017906; doi:10.1371/journal.pone.0321499)
Supplement: S4 Table — Inter-quartile range (IQR). GK: Goalkeeper. FB: Full-back. CD: Central Defender. CM: Central Midfielder. WF: Winger Midfielder. CF: Central Forward. (DOCX) [file pone.0321499.s004.docx]

| **Position** | **Low Entry Speed(Mean ± SD, IQR) (95% CI)** | **Medium Entry Speed(Mean ± SD, IQR) (95% CI)** | **High Entry Speed(Mean ± SD, IQR) (95% CI)** | **Very High Entry Speed (Mean ± SD, IQR) (95% CI)** |
| --- | --- | --- | --- | --- |
| **GK** | 2.9 ± 2.1 (2.00) (−0.72 to 6.52) | 2.4 ± 1.8 (1.00) (−0.89 to 5.69) | 1.2 ± 0.5 (0.25) (0.26 to 2.14) | NA |
| **FB** | 9.0 ± 3.8 (4.00) (4.78 to 13.22) | 14.9 ± 7.7 (11.00) (5.20 to 24.60) | 2.6 ± 1.7 (1.50) (5.20 to 24.60) | 1.0 ± 0.2 (0.00) (0.62 to 1.38) |
| **CD** | 9.6 ± 3.7 (5.00) (5.65 to 13.55) | 12.5 ± 7.4 (6.00) (3.50 to 21.50) | 1.4 ± 0.7 (1.00) (0.34 to 2.46) | 1.0 ± 0.0 (0.00) (1.00 to 1.00) |
| **CM** | 11.7 ± 5.1 (6.25) (7.24 to 16.16) | 20.9 ± 14.4 (10.50) (9.28 to 32.52) | 2.5 ± 1.5 (2.00) (1.01 to 3.99) | 1.2 ± 0.4 (0.00) (0.61 to 1.79) |
| **WF** | 7.2 ± 3.7 (5.00) (3.25 to 11.15) | 11.3 ± 7.8 (6.75) (1.41 to 21.19) | 2.7 ± 1.9 (1.00) (0.42 to 4.98) | 1.1 ± 0.3 (0.00) (0.55 to 1.65) |
| **CF** | 10.0 ± 3.8 (3.50) (5.78 to 14.22) | 16.6 ± 7.8 (2.00) (6.71 to 26.49) | 2.4 ± 1.3 (2.00) (0.43 to 4.37) | 1.0 ± 0.0 (0.00) (1.00 to 1.00) |
| **Overall** | 9.0 ± 4.8 | 14.3 ± 11.1 | 2.3 ± 1.5 | 1.1 ± 0.3 |
