# Supplementary material for: Exploring turn demands of an English Premier League team across league and knockout competitions over a full season
Source: PLoS One. 2025 Apr 23;20(4):e0321499. doi: 10.1371/journal.pone.0321499 (PMC12017906; doi:10.1371/journal.pone.0321499)
Supplement: Attachment — Submitted filename: Response to Reviewers (2).docx [file pone.0321499.s007.docx]

Rebuttal document for: ‘**Exploring Turn Demands of an English Premier League Team Across League and Knockout Competitions Over a Full Season’** by Griffiths *et al.*

We would like to thank the reviewers for their comments on our work, which provided suggestions that have further improved the manuscript. We have responded to each of the reviewer comments below and where we have made a careful revision of the manuscript, these changes are tracked changes. If you have any questions regarding our rebuttal or revisions, please do not hesitate to contact us.

Reviewer #1:

**Dear Authors,
I would like to acknowledge the effort you have made, which has undoubtedly improved the readability and comprehension of the manuscript, particularly in the Results section. The data are no longer duplicated and now follow a logical order, which enhances clarity. I have, however, a few minor considerations:**

The authors thank the reviewer for taking their time to comment on and improve this manuscript.

**1. Introduction Section**

**While the authors describe the match demands in women’s soccer, why is there no mention of the demands in men’s soccer, given that this study was conducted with a male sample?**

The authors thank the reviewer for pointing out this error. This has been corrected and metrics from the same systematic review has been included but for male soccer players:

***Line 55-56:***

Linear demands such as high-speed and sprint running distances ranged from 618 to 1,001 m and 153–295 m, respectively, in professional male soccer players (64).

**I believe the first paragraph of this section should be restructured to follow the conventional approach for discussions. This means emphasizing the study’s objective and highlighting the main findings without referring to tables or supplementary materials. Please also review the first sentence of the following paragraph for clarity and relevance.**

We have redrafted the first paragraph of the discussion to rearticulate the objective of the study and present the key findings. We have done this without referring to tables or figures as advised. We have also redrafted the first sentence of the next paragraph to add clarity.

**Please see re-drafted paragraph below (line 305-318):**

The present study sought to determine the turn demands of soccer players in each playing position using data from the English Premier League, FA Cup, League Cup and Europa League of a single team. The primary findings showed no difference in turn demands between competition types (Premier League vs. knockout matches), but significantly greater total turns performed by CM (~35) compared to GK (~5), CD (~23) and WF (~21), and differences in turn characteristics between each position group for each angle and entry speed category.

In contrast to the present study’s findings, similar research from Dos’Santos *et al.* (17), showed CMs performed more turns on average (~38) compared to other positional groups (FB, CD, CF). Though CM performed more turns, the rationale for CM performing the most turns in both studies was shared; this was attributed to their duel attacking and defending roles.

**3. Competitions Categorization

Since the authors did not analyze turning movements separately for each competition but instead grouped regular season and knockout competitions together, should this not be explicitly stated throughout the manuscript (title and objectives for example)? There is potential for confusion when the title and objective suggest a distinction, but the data are not presented separately for these competitions.**

The authors thank the reviewer for this comment. We have acted upon the advice given and clarified this throughout the manuscript. Please see below the changes made to the title and within the manuscript:

**Title:**

~~‘An Exploratory Analysis Investigating the Turn Demands of the Premier League, FA Cup, League Cup and UEFA Europa League for an English Premier League Soccer Team’~~

‘Exploring Turn Demands of an English Premier League Team Across League and Knockout Competitions Over a Full Season’

**Line 123-126:**

Turning data were obtained from 49 match fixtures during 2022-23 season, from the Premier League (35 matches), UEFA Europa League (5 matches), League Cup (5 matches) and FA Cup (4 matches), the latter three competitions are grouped for analysis as ‘knockout’ competitions.

**Line 308:**

The primary findings showed no difference in turn demands between competition types (Premier League vs. knockout matches),

**Line 420-423:**

Despite these previous findings, the current study found no significant differences between turn characteristics when comparing knockout (FA Cup, League Cup and UEFA Europa League) vs. league soccer (Premier League).

**4. Table Numbering

Is it correct for the current Table 4 to retain this numbering, or should it be Table 2 due to the inclusion of supplementary material? Please verify and ensure consistency.**

The authors thank the reviewer for identifying this error. Line 299 now shows the table to be named, Table 2.

**5. GPS Accuracy

In the Introduction, the authors claim that GPS devices have a lower error margin than other tracking systems, such as LPS. Is this accurate? Could the authors confirm this with additional references to strengthen the statement?**

The authors have re-worded the following to provide a clearer and more accurate narrative for the reader:

**Line 96-99:**

These GPS systems, when evaluated against the gold-standard 3D-motion capture system, yielded lower error values than ~~than other tracking systems such as~~ video-based systems ~~and local positioning systems (LPS),~~ but lower validity than local positioning systems (LPS)(64), as well as often failing to quantify turn metrics (20).
