# Supplementary material for: Exploring turn demands of an English Premier League team across league and knockout competitions over a full season
Source: PLoS One. 2025 Apr 23;20(4):e0321499. doi: 10.1371/journal.pone.0321499 (PMC12017906; doi:10.1371/journal.pone.0321499)
Supplement: Attachment — Submitted filename: Response to Reviewers.docx [file pone.0321499.s006.docx]

Rebuttal document for: ‘**An Exploratory Analysis Investigating the Turn Demands of the Premier League, FA Cup, League Cup and UEFA Europa League for an English Premier League Soccer Team’** by Griffiths *et al.*

We would like to thank the reviewers for their comments on our work, which provided suggestions that have further improved the manuscript. We have responded to each of the reviewer comments below and where we have made a careful revision of the manuscript, these changes are tracked changes. If you have any questions regarding our rebuttal or revisions, please do not hesitate to contact us.

Journal Requirements:

**#1: Please ensure that your manuscript meets PLOS ONE's style requirements, including those for file naming.**

Formatting changes have been made where necessary.

**#2. Please note that funding information should not appear in any section or other areas of your manuscript. We will only publish funding information present in the Funding Statement section of the online submission form. Please remove any funding-related text from the manuscript.**

All funding statements have been removed except in the funding statement section.

**3. Please upload a copy of Figure 6, to which you refer in your text on page 25. If the figure is no longer to be included as part of the submission please remove all reference to it within the text.**

Apologies for this error. Figure 6 should have been Supplemental 2, please see changes in text or below:

~~“Fig 6~~ Supplemental 2 combines both turn angle and entry speed to highlight the high incidence rate of turns, which occurred over the season, between 125-130° and 3.40-3.60ms^-1^.”

**5. We notice that your supplementary figures are uploaded with the file type 'Figure'. Please amend the file type to 'Supporting Information'. Please ensure that each Supporting Information file has a legend listed in the manuscript after the references list.**

This error has been corrected.

**#Reviewer 1:**

**The study design is interesting and align with the scope of PlosOne, I believe it can provide useful information; however, different sections of the manuscript need to be corrected to a large extent.d. I will outline below point by point some suggestions for improvement, but the main issues that need to be addressed are as follows:**

**• Results section: It is disorganized, data is repeatedly presented in both tables and figures, and the significance levels are not included in the tables and figures.**

**• Stated objectives: The necessary methodology to address all of the objectives has not been applied, and the objectives themselves are not properly written.**

**• Writing of the discussion.**

Thank you for stating you found our research interesting and within the scope of PlosOne. The results section, objectives and discussion will all be addressed as per your following comments. Thank you for this feedback.

**Point by point:**

**Why do the authors refer to it as "COD" throughout the text, but use "Turn" in the title? I understand that this may be to avoid repetition of keywords, but it might be more appropriate to switch them.**

Thank you for highlighting this, we have now used the term ‘turn’ instead of COD throughout. There are two occasions we have used COD, to inform the reader of the interchangeable nature of these turns we have added the following line to the first paragraph of the introduction:

These turning metrics (the term turning is synonymous with change of direction [COD]; 25; 51; 17) are often overlooked….

**Why does the title include the term “significant”?**

We have removed the term significant from the title.

**Introduction Section**

**In the introduction, some aspects need to be more thoroughly justified. The authors conclude by stating they will use Sportlight’s® LiDAR technology and provide only a justification for its use, but this approach is not appropriate for the introduction. Instead of saying what they will use, the authors should describe the technologies available for measuring this aspect, their characteristics, and what factors need to be considered. The introduction is meant to establish context and provide a theoretical framework, not to describe the specific methodology that the authors will use.**

The following has been added to supply the reader with more context surrounding the rationale behind using LiDAR rather than any other tracking systems:

Tracking technology, from a sports science perspective, is used predominantly for external-load monitoring to reduce injury incidence rates and optimize performance (52). Daily use of this equipment allows for session-to-session adjustment of training periodisation to ensure that the training loads physically prepare players for the demands of a match (53; 54). The current industry-dominating tracking system in soccer is the global positioning system (GPS) (55). Since the in-competition legalization of GPS technology in 2015 by the International Football Association Board, it has been used by practitioners world-wide to provide on-pitch, external load metrics (55). It boasts benefits such as its relative affordability, its portable nature, and an instant feedback system; allowing for constant monitoring during both training and matches (53; 20). These GPS systems, when evaluated against the gold-standard 3D-motion capture system, yielded lower error values than other tracking systems such as video-based systems and local positioning systems (LPS)(20). However, with the recent integration of LiDAR technology, some of the current limitations faced by GPS, for example, satellite signals being blocked by stadiums/buildings, negative correlation between number of satellites signalling to the receiver and total distance and velocity measurement error, could lead to reduced usage in elite soccer. However, a validity study directly comparing LiDAR and GPS needs to be completed before any conclusions can be drawn from these assumptions.

**Additionally, I believe it is necessary to delve deeper into the quantitative demands of soccer, specifically in relation to COD.**

Thank you for your comment, we have added the following:

“Linear demands such as high-speed and sprint running distances ranged from 911 to 1,063 m and 223–307 m, respectively, in professional female soccer players (64). Whereas, turns have been reported at a higher variability, with recent literature reporting turns counts ranging from ~700 (6) to ~38 (17).”**Line 55: This is the first time the acronym "COD" appears.**

Full spelling has been written out.

“Change of direction (COD)”

**Lines 65-67: Please use the acronym correctly.**

The error lies in the abstract, where I have used the term “centre-backs”. This has been fixed.

**Line 61: The terms "Turn" and "COD" seem to be used interchangeably. Are they meant to refer to the same thing?**

As per the previous suggestion we have changed all references of COD to ‘turn’.

**Line 67: What is meant by “absence”?**

Thank you for your comment. In an effort to make this clearer, we have reworded the sentence to the following:

(removal of the start of the sentence which included the phrase ‘the absence of turn characteristics’). The biomechanical demands of these turns on the body are angle and velocity dependant (5,9–13). Therefore, quantifying the number of COD movements alone, lacks scientific purpose without the added context of entry speed and turn angle. Both factors influence the braking and propulsive force characteristics……..”

**Lines 93-100: The objectives need to be revised and not listed as bullet points. Additionally, the authors are not analysing the demands across different competitions, but rather within one team in the same competition. The last objective cannot be answered with the proposed methodology and is not addressed throughout the manuscript.**

This feedback has been addressed, and the following paragraph has replaced lines 93-100:

Building on the recent work of Dos’Santos et al. (17), this study aimed to use Sportlight’s® LiDAR technology to determine the turn demands experienced by soccer players in a professional team competing in the Premier League, UEFA Europa League, FA Cup and League Cup. Specifically, the study sought to analyze turn characteristics for each playing position (goalkeeper, central defender, full-back, central midfielder, wide forward, and center forward) and explore the relationships between different turn characteristics. Examine differences in turn demands between competitions (Premier League, FA Cup, League Cup, and UEFA Europa League), for a single team. Finally, this research aimed to provide insights practitioners can use to inform injury-prevention, rehabilitation, and return-to-play programs, though further validation of the findings is necessary before generalizing to normative data for wider application.

**Methods Section**

**Lines 108-131: Can the authors include values for the validity, reliability, and reproducibility of the methodology? The description currently only outlines the process but does not report its validity.**

The validity of the Sportlight system has been confirmed in Bampouras and Thomas (2022) validity study. We have added a sentence to the methods to say Sportlight has been assessed as a valid method of player performance monitoring (Bampouras and Thomas, 2022).

For football-specific movements (i.e., jogging, linear sprinting, curved sprinting, and turns of various angles), the system has been validated against gold standard three-dimensional motion capture systems (60). Trivial to small differences in time spent during different speed zones (d = 0.04–0.26) were observed, and root mean square error values of 0.04–0.014 m/s and 0.16–0.7 m/s2 for velocity and acceleration data, respectively.

Inclusion of full reference:

Bampouras, T. M. and Thomas, N. M. (2022) ‘Validation of a LiDAR ‑ based player tracking system during football ‑ specific tasks’, *Sports Engineering*. Springer London, 25(1), pp. 1–6. doi: 10.1007/s12283-022-00372-7.

**Lines 143-144: I do not understand the following statement: “If players had played in multiple positions, they were categorized into the position group in which they had played most matches.” Wouldn't it be more appropriate to categorize them based on the position they played in each match?**

We thank the reviewer for this comment, unfortunately, this is something that is a limitation of this research. Given the format in which the data was received we were not able to separate players from the position group they had been allocated to for the season. As stated in our study, we did not include any players that played less than 80% of their games in their allocated position group. Though this is not ideal, it is as close as we are able to get. We have added this in the limitations and hope the reviewer understands:

“In addition, each player was allocated a position group which was adopted for all matches. Although no players were included in our study that played less than 80% of their matches in their allocated position group, it is important to acknowledge that this is a limitation and future research should aim for 100% position-specific data”

**Line 144: The phrase "29 players played 80% of the matches" is unclear. Does this mean "No player had played less than 80% of their games in their allocated position group"?**

This has been altered to the suggested phrasing:

All players played >80% of their games in their allocated position group.

**Line 146: Please include a reference to justify that 85 minutes is considered a full session. Previous studies have used fewer minutes.**

Thank you for raising this, unfortunately, this assumption is based upon Sportlight’s algorithms rather than literature so we are unable to provide rationale. As this is the Sportlight’s pre-built threshold, we were unable to deviate from this. This has been identified as a limitation.

**Definition of significant turns: This needs to be more clearly and precisely defined.**

Thank you for highlighting this. We have re-worded the definition to the following:

‘A significant turn is postulated to elicit high biomechanical loads and emits the inclusion of low biomechanical load change of direction movements such as curvilinear running. Specific metrics which qualify a turn to be ’significant’ are as followed: a change of direction with a deceleration³ <-2 m/s2 upon entry to the turn, an angle change in direction of travel ≥20°, and a subsequent acceleration ≥2 m/s2 whilst exiting the turn, all within a 1 second duration. ‘

**Statistical analysis: Please explain the process of determining the interquartile range.**

This information has been added in the penultimate sentence of the statistical analysis section:

“The interquartile range (IQR) was calculated by subtracting the first quartile (25th percentile) from the third quartile (75th percentile) of the dataset.”

**Results:**

**Table 2 Title: There is an unnecessary period.**

Changed.

**Table 2: I believe the table could be formatted differently to improve comprehension. Instead of listing data sequentially, perhaps several values could be included within the same cell (in parentheses)? Why report both the mean and the median?**

A new table has been inserted. Here are the changes which have been made:

1. **Consolidation of Metrics**: Each cell now contains the mean ± SD followed by the IQR in parentheses for turn characteristics. For total turns, it includes the mean ± SD followed by the range (min–max).
2. **Removal of Medians**: As the reviewer questioned their necessity, they’ve been removed for clarity and brevity.
3. **Improved Layout**: Grouped data by positions and added column headings for better readability.

| **Position** | **Low Angle**  **(Mean ± SD, IQR)** | **Medium Angle**  **(Mean ± SD, IQR)** | **High Angle**  **(Mean ± SD, IQR)** | **Total Turns**  **(Mean ± SD, Min–Max)** |
| --- | --- | --- | --- | --- |
| **GK** | 1.44 ± 0.73 (1.00) | 1.57 ± 1.16 (1.00) | 3.59 ± 2.47 (2.00) | 4.77 ± 3.32 (1–15) |
| **FB** | 2.88 ± 1.69 (2.00) | 6.05 ± 3.50 (4.75) | 18.10 ± 7.65 (10.00) | 26.36 ± 10.32 (2–51) |
| **CD** | 3.03 ± 1.76 (2.00) | 5.47 ± 2.50 (3.00) | 15.03 ± 8.31 (6.00) | 22.82 ± 9.41 (1–54) |
| **CM** | 3.38 ± 1.96 (2.00) | 7.38 ± 3.48 (5.00) | 23.86 ± 15.72 (11.00) | 34.52 ± 17.93 (3–102) |
| **WF** | 2.48 ± 1.56 (2.00) | 4.40 ± 2.36 (3.00) | 14.38 ± 8.98 (8.75) | 20.70 ± 11.18 (2–68) |
| **CF** | 1.71 ± 0.95 (1.50) | 7.00 ± 1.15 (1.50) | 20.57 ± 3.51 (5.50) | 29.83 ± 4.49 (25–37) |
| **Overall** | 2.92 ± 1.79 | 5.82 ± 3.35 | 16.84 ± 12.14 | 24.50 |

**Line 124: Should this reference be to Table 2 or Table 3?**

Corrected.

**What is the difference between Table 1 and Figure 1, apart from expressing the same data in different formats? In my opinion, this is redundant. Perhaps only the figures should be included. Please also consider my earlier comments regarding Table 2.**

Thank you for pointing this out, we have removed the tables from the main text and will include the improved version of the table (as per your above statements) as a supplementary material.

**Line 203: The authors state “No significant differences were found between outfield players (p > 0.05) (Fig 2d),” but then report differences between positions.**

Apologies, this is a mistake as it was supposed to be referring to just low-angled turns. This has now been corrected:

No significant differences were found between outfield players for low angled turns (*p* > 0.05) (Fig 2d).

**The Results section needs a complete revision.**

We apologise to the reviewer for the unclear results section. We have separated and addressed the rest of this comment:

**It is not well organized, as the authors first present all the tables and figures, then repeat each one.**

We have removed the summary paragraph at the beginning of the results section whereby all figures and tables were initially introduced.

**Additionally, figure captions appear within the text, the narrative jumps from one table to another, and then back again.**

All figure captions have been made to be clearly not within text by adding a textbox around each caption. Additionally, all references to Table 2 are before all references to Table 3.

**Lines 260-261: I believe the heading is not appropriate for the content that follows.**

We understand your issues with this heading and have changed it to:

“Investigating how entry speed changes with turn angle”

**Why are there no indicators (simbols) in the tables and figures to help interpret significant differences?**

This was initially involved in the table, however, due to the number of significant differences there are between all of the position groups, the authors felt this caused more confusion rather than information. If this is something which is required, it can be included.

**Discussion Section:**

**Lines 308-318: The writing and composition of the first paragraph need improvement. Follow the order of the objectives when reporting the main findings. It is not correct to state, “The aim was to build upon research by Dos’Santos et al. (17)”—the objectives are different. Avoid referring to tables and figures. Instead, state the key contributions of the study.**

We have aimed to resolve this through removed the sentence referred to and re-structuring the surrounding sentences:

~~“The aim was to build upon research by Dos’Santos~~ *~~et al.~~*~~(17)~~~~.~~ Findings differed to similar research such as Dos’Santos *et al.*(17).~~, for example,~~ ~~t~~Their CMs performed more turns on average (~38) compared to other positional groups (FB, CD, CF).”

References to tables and figures have been removed as advised.

**Line 320: If the authors mention there are large discrepancies, they need to report those discrepancies clearly, providing their own quantitative values and those of the references they are comparing against.**

Thank you for this feedback. We have included this information in the following sentences:

“This can be seen in the variance in results with total average turns per player being ~726(7), ~304(6), ~183(26) compared to the current finding of ~24.”

**Lines 338-340: The authors merely restate the results without explaining, debating, or discussing them. This needs to be expanded upon.**

The following sentence has been added to provide the reader with some context of the importance of the results:

“This understanding of positional-based differences of turn angles allows practitioners to tailor their drills for players based on their position to ensure they are prepared for the demands of the match.”

**Avoid referring to tables and figures in the discussion section by merely restating previously mentioned results.**

The following sentence has been removed:

~~Equally, Fig 4 highlights the low incidence rates of turns completed at a low angle and high/very high entry speed.~~

**Line 365: Please review the format of the final citation.**

Changed.

**What are the practical applications of this study? Could practitioners use these results to create a player profile**

The authors thank the reviewer for this comment.

We have added in a comment to further highlight to readers that this information should be used for drill design to help ensure players meet the demands of matches:

“This understanding of positional-based differences of turn angles allows practitioners to tailor their drills for players based on their position to ensure they are prepared for the demands of the match.”

**Reviewer #2:**

**An Exploratory Analysis Investigating the Significant Turn Demands of the Premier League, FA Cup, League Cup and UEFA Europa League for an English Premier League Soccer Team.**

**Thank you for the opportunity to review this manuscript, which aimed aimed to investigate the relationship of aerobic capacity to spinal curvature and mobility in young soccer players. In this sense, it appears that the study design was well-thought out and accurately replicates what could conceivably be implemented in practice. In addition, the paper mentioned an important topic that provides in handball referees. However, I have certain doubts in the introduction, the design and also in results and discussion.**

The above comment does not relate to this manuscript.

**The abstract succinctly summarizes the study's objectives, methods, and key findings. It effectively communicates the significance of change of direction (COD) movements in soccer and their potential implications for player performance and injury risk.**

**1. The abstract should be indicated at the end as well as in the conclusions section some directions about these retrospective and perspectives futures.**
**2. The results need to be better explained.**

We thank the reviewer for their feedback, we have made the following changes to our results section within the abstract. This was done with the aim of simplifying this section to just a description of the findings:

“Primary findings show, on average, per match, CM performed more total turns (~35~~vs~5-30~~), ~~low ES turns (~12vs~3-10), medium ES turns (~21vs~2-17) and high angled turns (~24 vs ~4-21)~~ than all other playing positions. In addition, CM performed more low and medium entry speed and high angled turns than other outfield positions. There were no significant differences between turn frequencies and turn characteristics in different competitions (p >0.05).”

**Introduction**

**The introduction provides a solid background on the importance of COD in soccer, citing relevant literature to establish the context. It outlines the research gap the study aims to address, specifically the lack of comprehensive data on turning demands across different competitions and player positions.**

The authors thank the reviewer for their positive comments in response to our introduction.

**1. More information is needed to understand the background of the research topic. The introduction must be rewritten, since it is insufficient**

In response to both the present reviewer and reviewer 1, the introduction has been rewritten and more background added on tracking technology and turns as requested. The authors hope this has improved the level of detail for the introduction. We have re-attached new paragraphs below:

“Tracking technology, from a sports science perspective, is used predominantly for external-load monitoring in an effort to reduce injury incidence rates and optimize performance (52). Daily use of this equipment allows for session-to-session adjustment of training periodisation to ensure that the training loads physically prepare players for the demands of a match (53; 54). The current industry-dominating tracking system in soccer is the global positioning system (GPS) (55). Since the in-competition legalization of GPS technology in 2015 by the International Football Association Board, it has been used by practitioners world-wide to provide on-pitch, external load metrics (55). It boasts benefits such as its relative affordability, its portable nature, and an instant feedback system; allowing for constant monitoring during both training and matches (53; 20). These GPS systems, when evaluated against the gold-standard 3D-motion capture system, yielded lower error values than other tracking systems such as video-based systems and local positioning systems (LPS)((20). However, with the recent integration of LiDAR technology, some of the current limitations faced by GPS, for example, satellite signals being blocked by stadiums/buildings, negative correlation between number of satellites signalling to the receiver and total distance and velocity measurement error, could lead to reduced usage in elite soccer. However, a validity study directly comparing LiDAR and GPS needs to be completed before any conclusions can be drawn from these assumptions.”

**2. The literature must be updated and also take into account the last research carried out in the last five years.**

Thank you for this comment. We have added the below studies into the introduction:

Alanen, A.M., Gibson, E.S., Critchley, M., Benson, L.C., Jordan, M.J., Ferber, R. and Pasanen, K., 2023. Differences in situational patterns during change of direction movements greater than 90 in youth male and female soccer players. *Journal of Human Kinetics*, *89*, p.149.

Martínez-Hernández, D., Quinn, M. and Jones, P., 2023. Linear advancing actions followed by deceleration and turn are the most common movements preceding goals in male professional soccer. *Science and Medicine in Football*, *7*(1), pp.25-33.

van den Tillaar, R. and Uthoff, A., 2024. Acute effects of different loading protocols upon performance and kinematics of 180 degrees change of direction. *Sports Biomechanics*, pp.1-15.

**3. Please improve the last paragraph and connect the background section with the relation with the aims at the end.**

This was also identified as an area for improvement by Reviewer #1 therefore please see earlier comments surrounding the changes to the final paragraph. We have attached a repeat of the new paragraph below:

“Building on the recent work of Dos’Santos et al. (17), this study aimed to use Sportlight’s® LiDAR technology to determine the turn demands experienced by soccer players in a professional team competing in the Premier League, UEFA Europa League, FA Cup and League Cup. Specifically, the study sought to analyze turn characteristics for each playing position (goalkeeper, central defender, full-back, central midfielder, wide forward, and center forward) and explore the relationships between different turn characteristics. Examine differences in turn demands between competitions (Premier League, FA Cup, League Cup, and UEFA Europa League), for a single team. Finally, this research aimed to provide insights practitioners can use to inform injury-prevention, rehabilitation, and return-to-play programs, though further validation of the findings is necessary before generalizing to normative data for wider application.”

**Methods**

**The study employs a longitudinal within- and between-subject comparative design, which is appropriate for analyzing turn metrics across different competitions and player positions. The use of Sportlight®’s LiDAR tracking system for data collection is a strength, as it allows for high-resolution tracking of player movements. The methodology is well-detailed, including the setup for home and away matches, which enhances the reproducibility of the study.**

The authors thank the reviewer for this comment.

**1. Sample. Can you add in method section a paragraph with the sample size calculated with G-power? Can you add information that have relationship with studies utilized in the introduction. Include Sample size calculation with G*Power (**[**www.gpower.hhu.de**](https://www.gpower.hhu.de)

The authors have included their sample size calculations as follows:

“Using Dos’Santos et al., (2022) effect size (0.19) a calculation was completed on G*Power (p=0.05, power=0.8). Results from these calculations concluded a minimum of 40 subjects was the necessary sample size. However, a post-hoc power calculation using the current studies effect size (0.41) found a sample size of 22 was needed for a power of 0.95 (p=0.05), showing our sample size to be sufficient.”

**2. Where is the inclusion or exclusion criteria? Incomplete**

Due to the nature of the data collection for this research there isn’t an exclusion criterion as such. The only restriction is during match average analysis where players who played less than 85 minutes (this is detailed in the sentence below) were excluded. Other than this, all turns which occurred on the pitch which aligned with our ‘significant turn’ definition, were included in analysis. All players were from the same team.

“Analysis which considered per-match averages only included turn data from players who played >85 minutes as this is considered a ‘full-session’ by Sportlight®, thus, substitutes were not considered. Analysis which looked at individual turn characteristics, i.e., average entry speed (ms^-1^) for each turn category, considered all turns, regardless of the athletes playing time.”

**3. The table 1. The The definition of each subcategory must be explained much better, and the values given in other columns**

The authors thank the reviewer for their comment. Here is the amended table:

| **Sub-Category/Turn Characteristic** | **Definition** |
| --- | --- |
| Low Entry Speed | When entering the turn, the instantaneous speed of the player upon initiation of the deceleration (when the players deceleration exceeded ≤−2 m/s^2^) is considered to be ‘low’. (<3.0ms^-1^) |
| Medium Entry Speed | When entering the turn, the instantaneous speed of the player upon initiation of the deceleration (when the players deceleration exceeded ≤−2 m/s^2^) is considered to be ‘medium’. (3.0-5.5ms^-1^) |
| High Entry Speed | When entering the turn, the instantaneous speed of the player upon initiation of the deceleration (when the players deceleration exceeded ≤−2 m/s^2^) is considered to be ‘high’. (5.5-7.0 ms^-1^) |
| Very High Entry Speed | When entering the turn, the instantaneous speed of the player upon initiation of the deceleration (when the players deceleration exceeded ≤−2 m/s^2^) is considered to be ‘very high’. (>7.0 ms^-1^) |
| Low Angle | The angle of the turn (see definition below) was 20- 59° |
| Medium Angle | The angle of the turn (see definition below) was 60-119° |
| High Angle | The angle of the turn (see definition below) was 120-180° |
| Entry Speed | The instantaneous speed of the player upon initiation of the deceleration (when the players deceleration exceeded ≤−2 m/s^2^) (17) |
| Turn Angle | The angle of the turn was computed as the angle between the acceleration and deceleration vector in the horizontal plane based on the estimated whole-body centre of mass (estimations were based on cluster points used to localize players by proprietary software) (17,20) |

**Result**
**The results are presented clearly, with a focus on the average number of turns performed by different player positions. The findings indicate that central midfielders (CM) perform significantly more turns compared to other positions, which is a valuable insight for coaches and trainers. The categorization of turns based on angle and entry speed provides a nuanced understanding of player movements.**

The authors thank the reviewer for these comments.

**1. Please follow the instructions for the review when presenting tables and figures.**

- Corrections have been made.
  **2. Add information about 95% CI, upper and lower.**

We have added a column for 95% CI to both tables, these can be found in the supplementals as per reviewer #1’s recommendations.

**Discussion**
**The discussion effectively interprets the results in the context of existing literature. It highlights the implications for position-specific training and rehabilitation programs, emphasizing the need for tailored approaches based on the unique demands of each position. The authors also acknowledge the limitations of the study, such as the focus on a single team, which could affect the generalizability of the findings.**

The authors thank the reviewers for their positive comments for the discussion.

**Conclusion**
**The conclusion summarizes the key findings and their relevance to soccer training and injury prevention. It reinforces the importance of understanding turn demands to inform training strategies and return-to-play protocols.**

**The article is well-structured and provides valuable insights into the turning demands of elite soccer players. The use of advanced tracking technology and a robust methodological approach strengthens the findings. However, future research could expand on this work by including a larger sample size and examining additional contextual factors such as player age and sex.**

The authors thank the reviewer for their comments on our conclusion.

**References**
**The article cites relevant studies and literature, which supports the claims made throughout the text. This enhances the credibility of the research and situates it within the broader field of sports science.**
**In summary, this study contributes significantly to our understanding of the physical demands placed on soccer players during matches and offers practical applications for training and rehabilitation.**

The author values these comments and thanks the reviewer for taking their time to comment on our research.
